# Diagnostic, Structured Classification and Therapeutic Approach in Cystic Pancreatic Lesions: Systematic Findings with Regard to the European Guidelines

**DOI:** 10.3390/diagnostics13030454

**Published:** 2023-01-26

**Authors:** Christopher Kloth, Benedikt Haggenmüller, Annika Beck, Martin Wagner, Marko Kornmann, Jochen P. Steinacker, Nora Steinacker-Stanescu, Daniel Vogele, Meinrad Beer, Markus S. Juchems, Stefan A. Schmidt

**Affiliations:** 1 Department of Diagnostic and Interventional Radiology, Ulm University Medical Center, Albert-Einstein-Allee 23, 89081 Ulm, Germany; 2 Institute of Pathology, Ulm University Medical Center, Albert-Einstein-Allee 23, 89081 Ulm, Germany; 3 Department of Internal Medicine 1, Ulm University Medical Center, Albert-Einstein-Allee 23, 89081 Ulm, Germany; 4 Department of General and Visceral Surgery, Ulm University Medical Center, Albert-Einstein-Allee 23, 89081 Ulm, Germany; 5 Department of Diagnostic and Interventional Radiology, Konstanz Hospital, Mainaustraße 35, 78464 Konstanz, Germany

**Keywords:** cystic pancreatic lesions, intraductal papillary mucinous neoplasm (IPMN), mucinous cystic neoplasm (MCN), serous cystic neoplasm (SCN), solid pseudopapillary neoplasm (SPN)

## Abstract

Due to the increasing use of cross-sectional imaging techniques and new technical possibilities, the number of incidentally detected cystic lesions of the pancreas is rapidly increasing in everyday radiological routines. Precise and rapid classification, including targeted therapeutic considerations, is of essential importance. The new European guideline should also support this. This review article provides information on the spectrum of cystic pancreatic lesions, their appearance, and a comparison of morphologic and histologic characteristics. This is done in the context of current literature and clinical value. The recommendations of the European guidelines include statements on conservative management as well as relative and absolute indications for surgery in cystic lesions of the pancreas. The guidelines suggest surgical resection for mucinous cystic neoplasm (MCN) ≥ 40 mm; furthermore, for symptomatic MCN or imaging signs of malignancy, this is recommended independent of its size (grade IB recommendation). For main duct IPMNs (intraductal papillary mucinous neoplasms), surgical therapy is always recommended; for branch duct IPMNs, a number of different risk criteria are applicable to evaluate absolute or relative indications for surgery. Based on imaging characteristics of the most common cystic pancreatic lesions, a precise diagnostic classification of the tumor, as well as guidance for further treatment, is possible through radiology.

## 1. Introduction

The rate of incidentally detected cystic pancreatic lesions in everyday diagnostic routines is continuously increasing. The reasons for this are the ongoing technical advances in ultrasound, computed tomography (CT), and magnetic resonance imaging (MRI). In particular, due to higher resolutions, more and smaller, previously undetectable findings are being described. Furthermore, the number of cross-sectional diagnostic examinations in daily clinical routine is also rising steadily, resulting in an increased possibility for the incidental detection of small findings. In addition, the increased aging of society, with the age of patients to be examined constantly rising, is of importance [1,2].

After detecting the lesion, the next step is usually to determine the precise description, the diagnostic classification, and the best course of action, including recommendations for further diagnosis or therapy. Pure cystic lesions of the pancreas have to be differentiated from tumors with solid and cystic components [3]. The prevalence of incidental pancreatic cystic findings in asymptomatic patients varies from 2–3% in CT examinations [4] and up to 20% in MRI examinations [5,6]. The overall precision (accuracy) of the detection and specific assignment of pancreatic neoplasia is higher in MRI (with 40-95%) than in CT (with 40–81%), according to expectations [4]. The guidelines favor MRI as the preferred imaging modality; CT is recommended for detecting calcifications of the parenchyma in the context of pancreatitis or in the presence of mural or central calcifications [7]. Furthermore, CT is indicated in the simultaneous presence of pancreatic carcinoma or its recurrence (grade II C recommendation for both cases) [7]. In addition to radiological cross-sectional imaging, endoscopic ultrasound (EUS) plays an important diagnostic role due to its high spatial resolution [8].

Of the cystic lesions detected, a majority are benign, although some of the lesions have a risk of transformation: Up to 30% of the detected cystic lesions are classified as malignant, and up to 30% of others are classified as potentially malignant [9]. Precise identification of the malignant potential is of crucial importance for further therapeutic approaches. Some cystic pancreatic lesions follow an adenoma–carcinoma sequence in a complex manner and may fluently transform into a malignant form [10]. A structured analysis of the findings, for example, according to the template by Persigehl [11], is helpful for a specific classification.

The aim of our review is to summarize the most common cystic pancreatic lesions, describe their specific imaging characteristics, and provide some guidance for further management.

## 2. Systematic Classification

The morphologic classification of cystic pancreatic lesions detected incidentally on CT or MRI is based on the histologic subtypes.

The first step toward the correct diagnosis of a cystic pancreatic tumor is a morphologic description that is as accurate as possible. Therefore, analogous to the Bosniak classification of renal cysts, strategies for the systematic assessment of cystic pancreatic lesions have been established in recent years [12,13,14]. Generally, four morphological subtypes are distinguished:

- Unilocular cysts without solid parts, septa, or calcifications
- Macrocystic tumors, cysts usually > 1–2 cm (oligocystic or multicystic)
- Microcystic tumors, cysts usually < 1 cm
- Cystic tumors with solid parts

This is in relation to the histologic entity classification: Serous cystadenomas (SCA), mucinous cystic neoplasms (MCN), and intraductal papillary mucinous neoplasms (IPMN) represent the vast majority of all primary cystic pancreatic neoplasms (approximately 90%) [15]. The remaining small group mainly represents rare cystic neoplasms as well as solid lesions with cystic transformation; true epithelial cysts of the pancreas are a rarity. An overview of the most important entities is shown in Table 1 [7,16,17,18,19]. The most common of these will be discussed as follows.

## 3. Most Frequent Tumor Entities

### 3.1. Unilocular Cystic Lesions

In the diversity of cystic lesions of the pancreas, unilocular cysts represent the largest group. Among them, pseudocysts are the most frequent, especially after an anamnestic history of pancreatitis or trauma. In this regard, it is important to consider the overall appearance of the pancreas with possible post-inflammatory or chronic inflammatory changes, such as partial atrophy or stippling calcifications.

**Figure 1:**
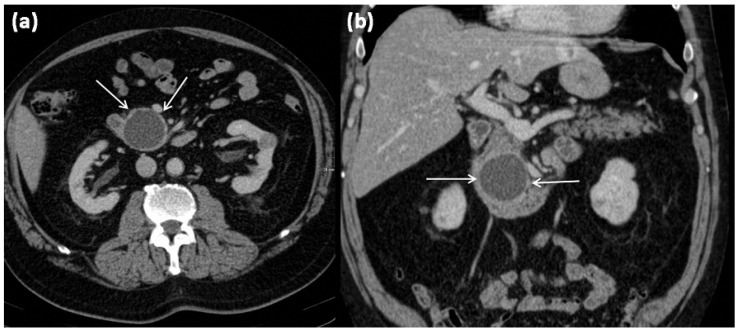
Axial (**a**) and coronal (**b**) contrast-enhanced computed tomography of the abdomen in venous phase in a 74-year-old male patient. In the processus uncinatus, a sharply demarcated pseudocyst (arrows) without solid components, septa, or surrounding infiltration can be seen. At most, the pseudocystic wall shows mild contrast enhancement.

**Figure 2:**
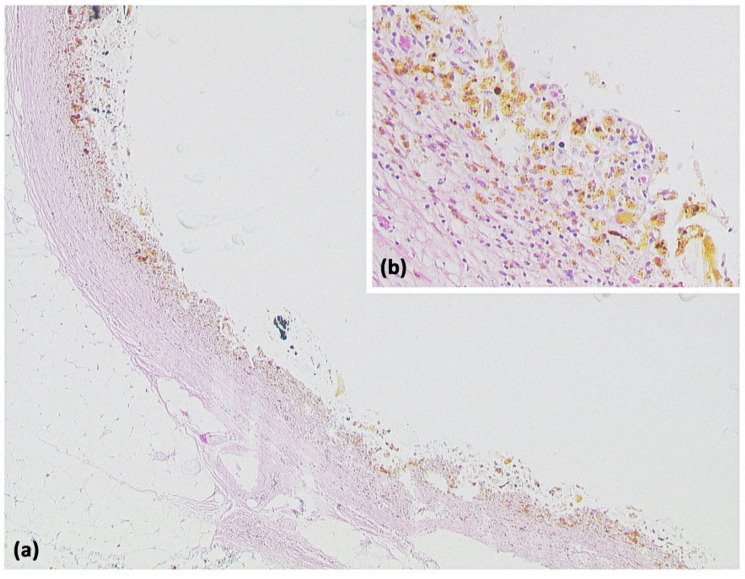
Pancreatic pseudocyst ((**a**) HE, original magnification 25:1). A pseudocyst located in the peripancreatic fat tissue with a fibrosed wall. At higher magnification ((**b**) HE, original magnification 100:1), it can be seen that no epithelial lining is present. Instead, the cavity is bordered by connective tissue with interspersed macrophages and lymphocytes. The visible yellowish pigment is most likely bile pigment.

Pseudocysts may be very large. No septa or solid components are present, and a subtle, contrast uptake of the wall is possible (Figure 1 and Figure 2). The presence of irregular wall thickening requires further investigation. An aspiration by means of endosonographically guided puncture is possible; alternatively, follow-up CT or MRI imaging can be used [12]. The guidelines recommend endosonography or cross-sectional imaging for cysts < 15 mm, and both procedures, including fine-needle aspiration, for cysts > 15 mm [7]. In the resulting punctate, pseudocysts contain pancreatic enzymes, such as amylase and lipase [20]. In a typical pseudocyst, there is no elevation of carcinoembryonic antigen (CEA) or CA 19-9 in the puncture specimen [21]. The resorption of the cyst within a few weeks after pancreatitis or trauma is possible, but with existing pancreatic secretion maintenance, persistence or even size progression may occur.

**Figure 3:**
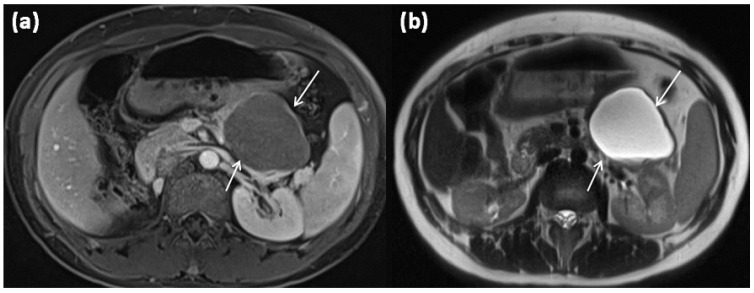
Axial MRI of a 12-year-old female patient with T1w sequence after gadolinium-based contrast administration (**a**) and T2w sequence (**b**). The pancreatic body shows a relatively large, sharply demarcated lesion compared to the surrounding parenchyma, which corresponds to a simple ductal cyst (arrows). No septa or nodular proliferations are detectable, nor are environmental reactions or infiltrative growth present.

**Figure 4:**
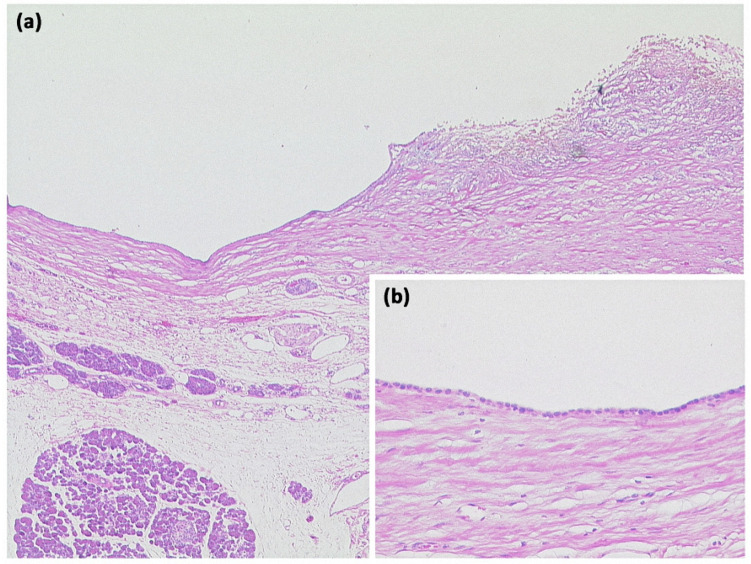
Simple ductal cyst ((**a**) HE, original magnification 25:1). Unilocular cyst with a slightly fibrosed wall and signs of trauma/rupture (upper right). Inset: The cyst is lined by a flat monolayer epithelium ((**b**) HE, original magnification 100:1).

Post-inflammation, the duct may also be dilated or consist of caliber variations and irregular borders. As a differential diagnosis, unilocular cysts may also be present after trauma (Figure 3 and Figure 4) [11].

### 3.2. Macrocystic Tumors: Mucinous Cystic Neoplasms (MCN)

**Figure 5:**
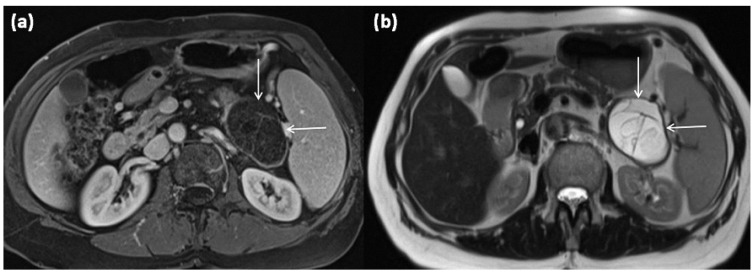
MRI of a 67-year-old female patient with T1w sequence after gadolinium-based contrast administration (**a**) and T2w sequence (**b**). A large multicystic mass of the pancreatic tail (arrows) with tiny septa in T2w, as well as mild contrast enhancement of each septa, is seen.

**Figure 6:**
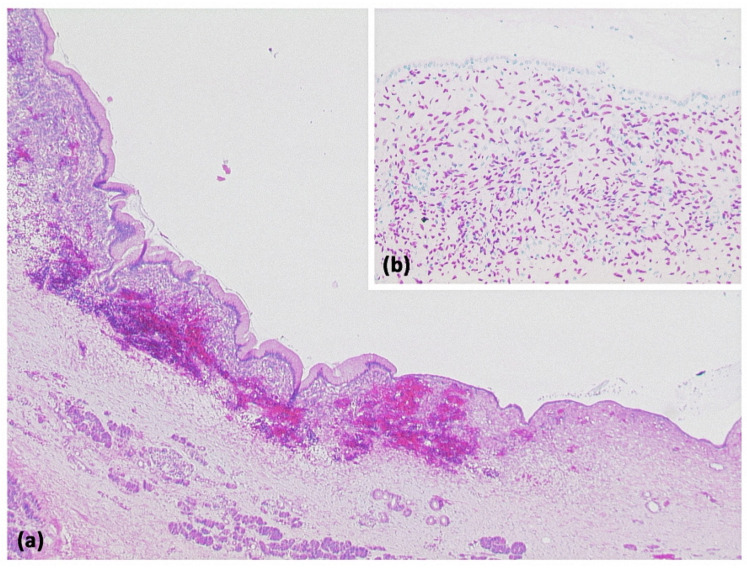
Mucinous cystic neoplasia ((**a**) HE, original magnification 25:1). A large unilocular cyst lined by mucinous and sometimes flattened epithelium. Subepithelial, a cell-rich stroma expressing the progesterone receptor, can be seen ((**b**) original magnification 100:1).

MCNs account for approximately 10% of cystic pancreatic tumors [10]. Mucinous cystic neoplasms in this context are solitary, well-circumscribed space-occupying lesions with smooth surfaces and septations, which appear monocystic or oligocystic and have a rather strong wall (Figure 5) [22,23]. The cyst content is mucinous and rich in diagnostically useful CEA and CA 19-9 [21]. In histopathology, ovarian stroma is typically found in MCN [21,24], which explains its almost exclusive occurrence in premenopausal women (Figure 6).

MCN has malignant potential and is, therefore, an indication for surgical resection [13,21]. According to current WHO nomenclature, a histological distinction is made between MCN with low-grade dysplasia, MCN with high-grade dysplasia, and MCN with associated invasive carcinoma [16]. The invasive component usually manifests as ductal adenocarcinoma, sometimes with the distinctive aspect of undifferentiated carcinoma with osteoclast-like giant cells. Interestingly, extracellular mucinous carcinomas are not associated with MCNs.

Size can vary widely, single sizes up to 35 cm have been described, with sizes greater than 10 cm being rare [13]. Surrounding the MCN is a fibrous pseudocapsule of variable thickness. In 95% of cases, the tumors are localized in the pancreatic body and pancreatic tail [13,21,23,25].

The often thick cyst walls are well-demarcated in any cross-sectional imaging. MRI usually shows a macrocystic mass, which is usually composed of individual parts often exceeding 2 cm [26]. The cyst content, typically with a T2w hyperintense signal, may be aqueous, but may likewise be hemorrhagic or have varying protein concentrations with varying degrees of thickening. Therefore, in CT, higher HU values, and in MRI, the variability of native T1 and T2 signals, can be observed. After contrast application, an enhancement of the cyst wall, as well as of the septations, is seen [25]. In this context, especially in dynamic MRI sequences, an enhancement can be observed, particularly in the later phases due to the fibrous cyst walls; in addition, small mural nodules are possible [26,27,28]. MRCP is mainly used for the differential diagnostic exclusion of a ductal connection, which is not present in MCN.

Peripheral eggshell-like calcifications, which are described as pathognomonic, are rare overall [13], but are easier to detect using CT and are considered to be a criterion for a potentially malignant transformation, especially in the case of new occurrence or particularly strong or widely peripheral manifestation [12]. An occurrence at an older age is suspicious for malignancy as well as large individual cysts of more than 6 cm and irregular and very thick cyst walls with papillary projections in the sense of solid nodular components or hypervascular areas [12,13,21,23].

The guidelines recommend surgical resection for MCN ≥ 40 mm, and also for symptomatic MCN or morphologic signs of malignancy, it is then recommended regardless of their size (grade IB recommendation) [7]. Cytologic workup by fine needle aspiration is recommended only if imaging is unclear and no differentiation between mucinous vs. non-mucinous neoplasm can be made (grade II C recommendation). Malignant potential can be determined this way [7].

### 3.3. Microcystic Tumors: Serous Cystic Neoplasms (SCN)

From the heterogeneous group of cystic pancreatic lesions, approximately 15% of all findings belong to serous cystic neoplasms (SCN), also known as serous cystadenoma. These can occur in all sections of the pancreas, with a slight preference for the pancreatic body and pancreatic tail [29]. Typically, patients aged 60–70 years old are affected. SCN may present with nonspecific upper abdominal symptoms, and the therapeutic procedure, in this case, depends on the intensity of the symptoms. There is no potential for malignant transformation in SCN.

Histologically, epithelial cells with a cubic shape and glycogen-rich cytoplasm are characteristic. No mucus production is found in this case. SCN shows no connection to the pancreatic duct system.

SCN can be subdivided into 3 subtypes, which differ in terms of their macroscopic aspect, localization, and age as well as sex distribution.

**Figure 7:**
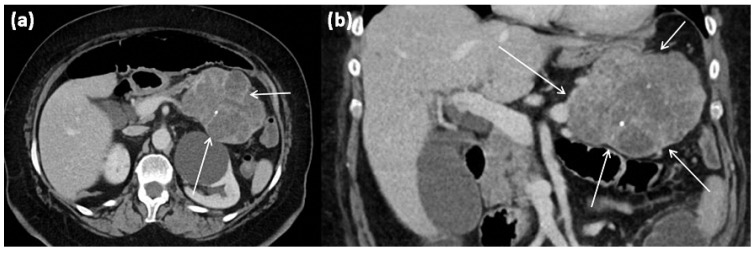
CT of a 63-year-old female patient (portal venous phase) in axial (**a**) and coronal (**b**) view. CT shows a large polylobulated tumor of the pancreas tail with partly cystic parts (arrows). In addition, single punctate calcifications and sharp demarcation to surrounding tissue can be seen.

**Figure 8:**
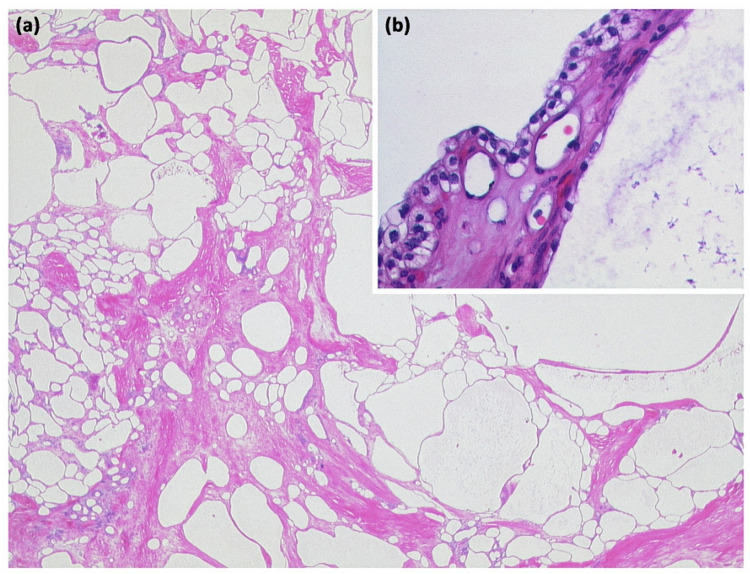
Serous cystadenoma with multiple compartments of different sizes ((**a**) HE, original magnification 12.5:1). The cavities are lined by a layer of atypia-free epithelia with clear cytoplasm ((**b**) HE, original magnification 200:1).

The most common type of SCN is serous microcystic adenoma (SCA). It is a relatively large (up to 25 cm, usually 6–11 cm), solitary, well-circumscribed tumor (Figure 7). It predominantly occurs in the body and tail. SCA presents a typical picture of honeycomb-like, arranged microcysts up to 2 cm, which are separated by delicate septa and do not communicate [30]. In the center of the lesion, a star-shaped scar is often found, which may show calcifications. This is presumably the fibrosed walls of a centrally located, collapsed cyst. The cysts appear fluid isointense, and the septa may show discrete contrast enhancement. The SCA appears most likely to arise from centroacinar cells. Immunohistochemically, the cells are positive for α-inhibin and for MUC1 and MUC6 (Figure 8). Women of older age are most frequently affected.

A rare subtype of SCN is serous-oligocystic adenoma (SOA), also known as serous-macrocystic adenoma. At this point, in contrast to SCA, a gender preference is seen in males, and usually, the tumors are localized in the pancreatic caput. The border of SOA is less well-defined compared to SCA, moreover, no central scar-like structure exists. Macrocystic SCN is presented with larger circumscribed cystic structures, which have been defined with a threshold > 2 cm in relation to individual cysts [31,32]. They may occur in combination with smaller cysts without a honeycomb-like aspect, which earned them the term oligocystic lesions. The microcysts are typically located in the center of the marginal macrocystic portions [33].

The third subgroup of SCN consists of lesions associated with von Hippel-Lindau syndrome (VHL-associated SCN). In this area, multiple SCNs with a multifocal appearance occur with a complete transformation of the pancreatic parenchyma [34]. In addition, there is an association with the occurrence of neuroendocrine tumors [35]. Asymptomatic patients with proven SCN should be followed up with once per year, after which, symptom-based follow-up is recommended (grade 2C recommendation) [7].

Once the diagnosis of SCN is confirmed, surgery is recommended only in patients with symptoms related to the compression of adjacent organs (grade 2 C recommendation) [7]. In approximately 60%, SCNs show size constancy, and size progression is documented only in approximately 40% [7].

### 3.4. Cystic Tumors with Solid Portions: Solid Pseudopapillary Neoplasms (SPN)

The solid pseudopapillary pancreatic tumor (Frantz tumor) is a rare epithelial tumor entity of the pancreas, which is responsible for less than 5% of cystic pancreatic tumors [29].

According to the current WHO classification [16], these tumors are principally classified as low-grade malignant. High-grade malignant transformation, which is associated with a much less favorable prognosis, may also occur. SPN usually occurs in young women in the 2nd and 3rd decades of life. Case reports in later decades of life have also been described in the literature [36,37]. There is a clustering in women of Asian or African descent; men are rarely affected.

**Figure 9:**
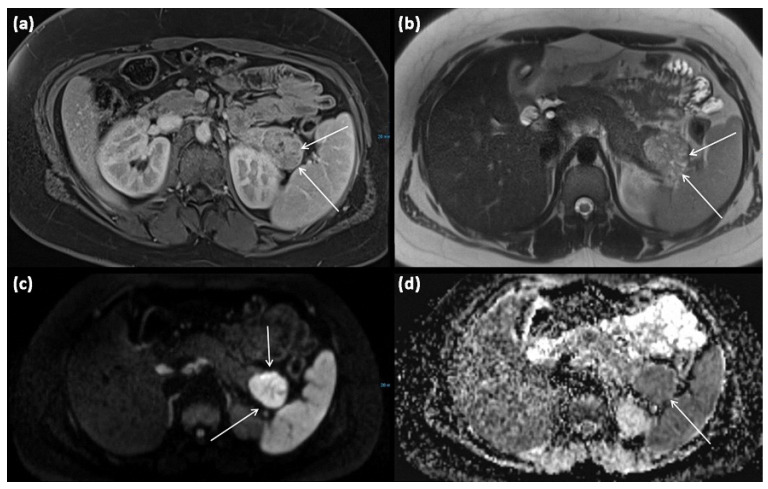
MRI of a 15-year-old female patient with axial T1w after gadolinium-based contrast administration (**a**) T2w (**b**) as well as DWI (diffusion-weighted imaging) weighting ((**c**), b = 1000) and ADC map (apparent diffusion coefficient) (**d**). MRI shows a solid tumor of the pancreatic tail. In T2w, almost exclusively, intermediate to hypointense signals can be seen, showing high cellularity in the absence of cystic parts. There is a strongly hyperintense signal in DWI with a signal decrease in ADC. The tumor is marked with arrows in all sequences.

SPN can be localized in all sections of the pancreas, within which, some studies show a slightly more common localization in the tail of the pancreas (Figure 9). The radiologic appearance is characterized by a large tumor and a mixture of cystic and solid portions. Smaller tumors are especially likely to appear completely solid. Denser internal portions may result from characteristic hemorrhages with hyperintense signals in T1. Central calcifications are detectable in up to 30% of the described cases [21].

The risk of malignant transformation, as well as the occurrence of distant metastases, have been described as 15% in the literature [38], and eventual metastases are most commonly found in the liver as well as in the peritoneum.

**Figure 10:**
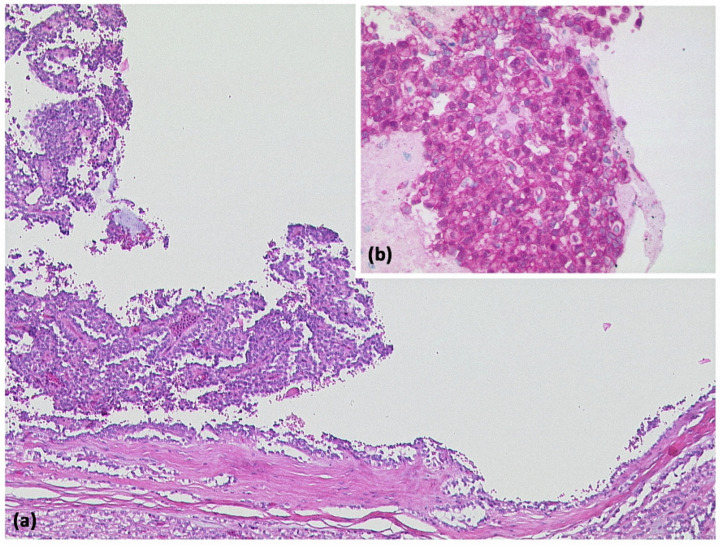
Solid-pseudopapillary neoplasia with cystic appearance and pseudopapillary structures ((**a**) HE, original magnification 50:1). Immunohistologically, cytoplasmic and nuclear expression of β-catenin is typical for this entity ((**b**) original magnification 200:1).

Histopathologically, SPN shows solid and pseudopapillary tissue portions, which are supported by vascular hyaline stromal strands and may be interspersed with hemorrhages (Figure 10) [20]. The tumor cells in this case are monomorphic with eosinophilic or bright cytoplasm. Classically, a mutation is present in the CTNNB1 gene, resulting in a defective β-catenin protein and associated with the immunohistochemically detectable nuclear and cytoplasmic expression of β-catenin.

The prognosis is very good with complete resection, with a cure rate of over 95% in several studies [39], although this is worse at older ages.

### 3.5. Intraductal Papillary Mucinous Neoplasms (IPMN)

IPMN represents the most common neoplasm of the pancreas with a cystic appearance, accounting for up to 25% of all lesions. IPMN originates from the main or an accessory duct of the pancreas and produces mucin [40]. There is a preference for localization in the pancreatic head, but a multilocular appearance within the entire pancreas is also common [41].

Morphologically, three subtypes can be differentiated: main duct IPMN, branch duct IPMN, and a mixed type. Here, circumscribed ductal dilatation of the pancreatic duct is defined as main duct IPMN. If the pancreatic duct is unremarkable and a dilated side duct exists at the same time, it is referred to as side-branch IPMN [13].

This has prognostic and therapeutic consequences: main duct IPMNs have a higher malignant potency, and invasive pancreatic carcinomas may develop from initially benign IPMNs [40]. Data here vary from 7% to 34% for carcinoma in situ and from 25% to 44% for invasive carcinoma.

High-risk factors for malignancy (so-called “high-risk stigmata”) are a circumscribed main duct dilatation to more than 10 mm without circumscribed obstruction, mural solid nodules (“papillary projections”) with contrast enhancement, and the obstruction of the bile duct [42]. In contrast, main ductal dilatation to 5–9 mm, thickened or contrast-enhancing cyst walls, mural nodules without contrast enhancement (best delineated in T2w weighted sequences), lesion sizes greater than 3 cm, abrupt main ductal termination, or associated lymphadenopathy are considered precursors or “worrisome features” [40,43,44].

As a clinical criterion, several studies have found that the presence of icterus has a positive predictive value for malignancy [7]. This fact fits with the obstruction of the bile duct described above. Several studies have also recently identified an elevated serum level of CA 19-9 as an independent predictor of malignancy in IPMN [7,45,46,47].

Histologically, several subtypes of IPMN can be contrasted: gastric-foveolar, villous-intestinal, and pancreaticobiliary subtypes are distinguished. However, these cannot be differentiated from each other morphologically in images.

#### 3.5.1. Main Duct IPMN

Main duct IPMN is characterized by circumscribed dilatation of the main pancreatic duct. This may result in cystic-impressive formations favoring the head of the pancreas. The expansion of the viscous secretion may lead to an overestimation of the tumor volume, and there is always an abrupt short transition zone to unaffected ductal segments [21].

Surgical therapy is recommended for main duct IPMN; there are no randomized trials comparing surgery with follow-up only [7].

#### 3.5.2. Branch Duct IPMN

**Figure 11:**
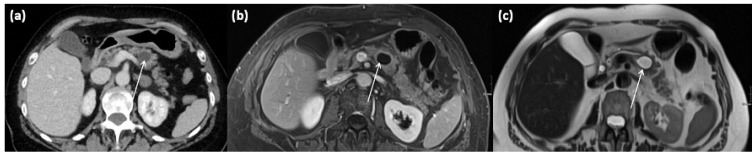
Axial CT in portal venous phase (**a**) and axial MRI with T1w after gadolinium-based contrast administration (**b**) as well as T2w native (**c**) in a 64-year-old female patient. In the pancreatic body, a sharply demarcated, roundly configured, cystic lesion is present, fit to a branch duct IPMN (arrows). There are no main duct dilatation, solid parts, or vegetations. T2w shows a homogenous hyperintense signal.

**Figure 12:**
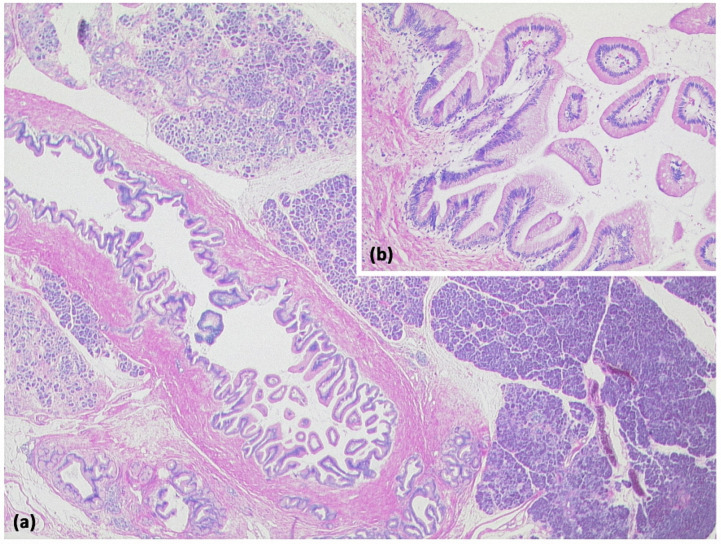
Intraductal papillary mucinous neoplasm (IPMN, (**a**) HE, original magnification 12.5:1). Papillary proliferate of mucinous cells in a dilated pancreatic duct. Higher magnification clearly shows gastral differentiation, with no evidence of high-grade dysplasia ((**b**) HE, original magnification 100:1).

Changes occur within the side branches of the pancreatic duct (Figure 11 and Figure 12), often in the area of the uncinate process. In many cases, multiple branch ducts are affected in the sense of a multifocal IPMN, which is present in up to 30% of cases [13]. In this case, each individual cystic lesion must be analyzed separately on imaging for signs of malignancy (grade 2C recommendation) [7]. Typically, a flask-like or grape-like configuration (“grape-like appearance”) is seen. It is often difficult to differentiate a solid tumor formation due to the only small size of an IPMN. To differentiate lateral duct IPMN from other mucinous tumors, such as MCN, evidence of a ductal connection should be obtained by MRCP or ERCP.

**Figure 13:**
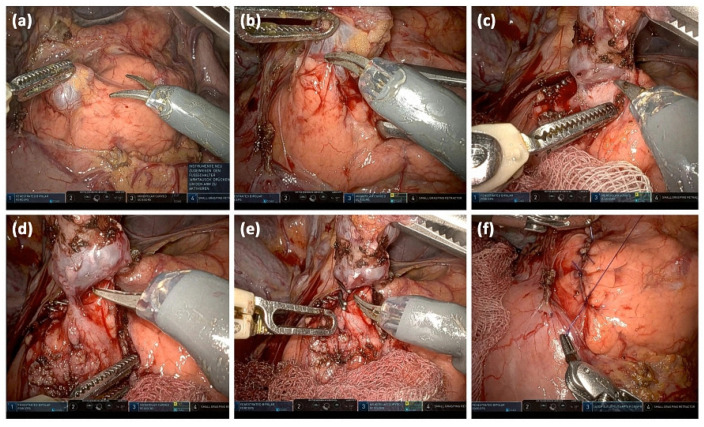
Intraoperative images of a robotically assisted IPMN resection: Opening of the bursa omentialis via the omentum minus and identification of the cystic lesion; the start of preparation after incision of the pancreatic capsule (**a**). Stepwise preparation of the pancreatic parenchyma from the lesion (**b**). Complete separation of the cystic lesion from the pancreatic parenchyma (**c**). Visualizing the connection to the pancreatic main duct (**d**). Clipping of the connection to the main duct and transection (**e**). Readaptation of the pancreatic capsule (**f**).

Follow-up intervals in the absence of worrisome features are dictated by the maximum sizes of IMPN: <10 mm (every 12 months), 10–20 mm (every 6–12 months), and 20–30 mm (every 3–6 months). Alternatively, direct resection can always be performed [40]. In young patients, the risks of malignancy and surgery must be weighed against the burden of lifelong follow-up [7]. In this context, the various surgical procedures must be considered. Presently, robotic, minimally invasive surgical approaches are introduced in trying to decrease surgical risks (Figure 13).

**Figure 14:**
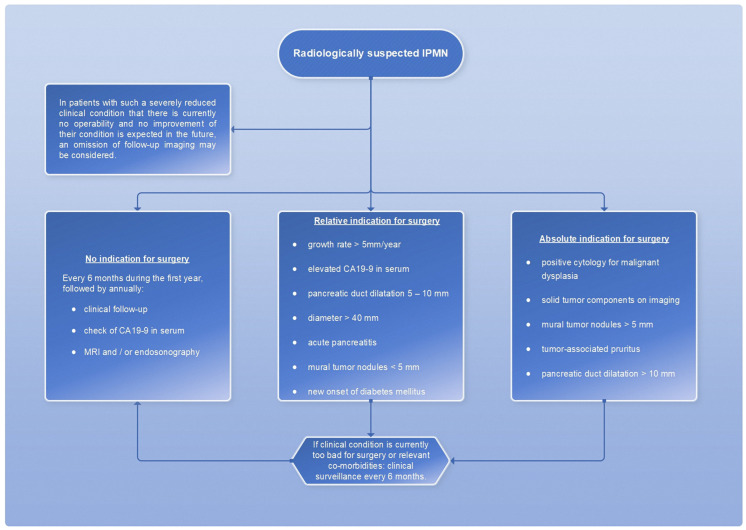
Flowchart for the therapeutic procedure in case of IMPN, adapted from [7].

However, a cyst size ≥ 30 mm without other radiologic or clinical risk factors has been described to have a positive predictive value for malignancy ranging from 27% to 33% [7]; therefore, cyst size alone is not an appropriate measurement of indication for surgery. The absolute and relative surgical indications are summarized in a scheme (Figure 14).

#### 3.5.3. Mixed Type

**Figure 15:**
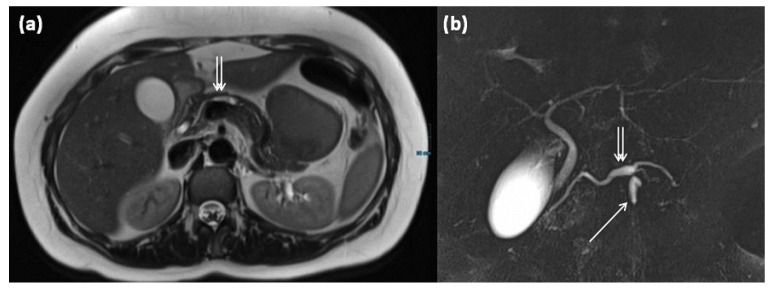
MRI of a 39-year-old female patient with T2w (**a**) and magnetic resonance cholangiopancreaticography (MRCP) (**b**). Images show a clear demarcated main duct dilatation in terms of a main duct IPMN (double arrow). Furthermore, the extension of a branch duct (arrow) can be seen, thus of a combined main and branch duct IPMN is present (mixed-type).

In addition to the two most common types, the mixed type is delineated as a third entity; this has ductal extensions of both main and branch ducts (Figure 15). In spite of that, the mixed type has the same risk of malignant transformation as the main duct type, so surgery is recommended (grade 2C recommendation) [7].

## 4. Differential Diagnostic Difficulties, Special Types, and Advanced Diagnosis

The differentiation of pancreatic cystic-like lesions presents some pitfalls. For example, the central scars of microcystic SCN may resemble a solid portion or the papillary projection of an IPMN. In this case, the evidence of ductal involvement of the IPMN is most important for differentiation.

**Figure 16:**
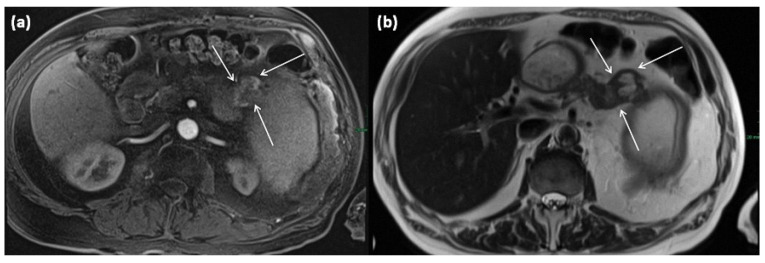
MRI of a 60-year-old patient with a partly cystic and partly solid mass of the pancreatic tail. Arterial T1w after gadolinium-based contrast administration (**a**) typical peripheral hyperarterilization (arrows) can be seen. In T2w, solid as well as cystic parts are present (**b**).

**Figure 17:**
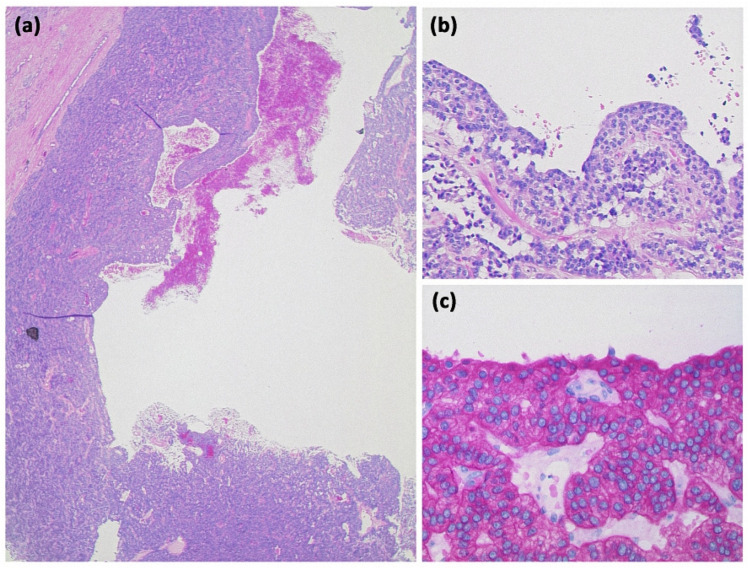
Cystic neuroendocrine tumor. In overview magnification ((**a**) HE, original magnification 12.5:1), a rather cell-dense tumor with a centrally located cystic cavity can be seen. At higher magnification ((**b**) HE, original magnification 100:1), the tumor consists of monomorphic cells with isomorphic roundish nuclei. Immunohistochemistry revealed strong expression of synaptophysin ((**c**) original magnification 200:1) and chromogranin (not shown).

The extremely rare variant of a solid SCN is a differential diagnosis to neuroendocrine tumors (NET) (Figure 16 and Figure 17). In this case, the diagnostic criterion was shown to be the contrast agent behavior, which typically does not show washout in NET and can be observed more frequently in solid SCN [30]. The contrast behavior of NET is variable; in unclear cases, EUS-guided biopsy should be weighed to exclude NET.

**Figure 18:**
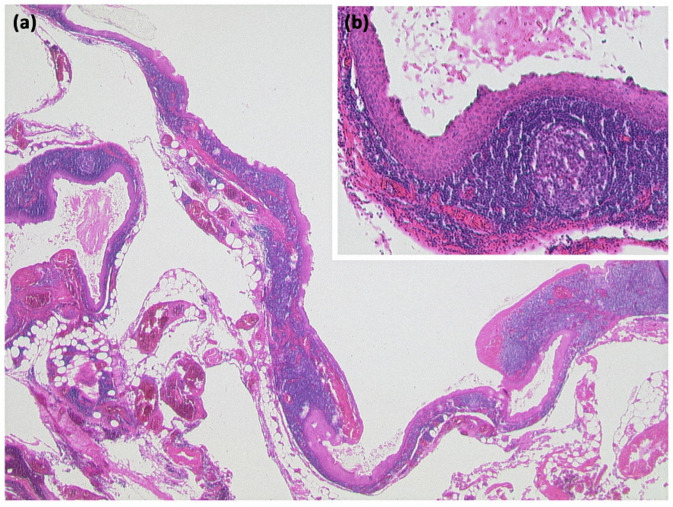
Lymphoepithelial cyst ((**a**) HE, original magnification 12.5:1): Overview magnification shows a cyst with several compartments and a cell-dense population in the cyst wall. Higher magnification reveals that the cysts are lined by an atypia-free squamous epithelium and accompanied by a lymphoid tissue with organoid structure and germinal centers ((**b**) HE, original magnification 50:1).

Mucinous cystic neoplasms, in contrast to oligocystic SOA, typically exhibit vigorous contrast enhancement of the cyst walls, which are usually much thicker [48]. Lymphoepithelial cysts of the pancreas represent a rare benign differential diagnosis of SCN with a very heterogeneous appearance (Figure 18) [49].

**Figure 19:**
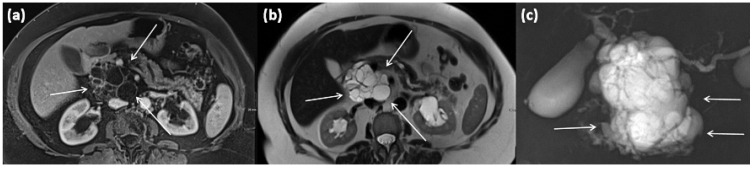
MRI of a 79-year-old female patient with axial T1w (**a**) after gadolinium-based contrast administration, T2w, and (**b**) magnetic resonance cholangiopancreaticography (MRCP) (**c**). A merging and communicating multicystic formation is seen at the head of the pancreas, with the single cystic parts showing marked differences in size. Septa show mild contrast enhancement. In T2w a homogenous hyperintense signal is present. The tumor is marked with arrows in all sequences.

**Figure 20:**
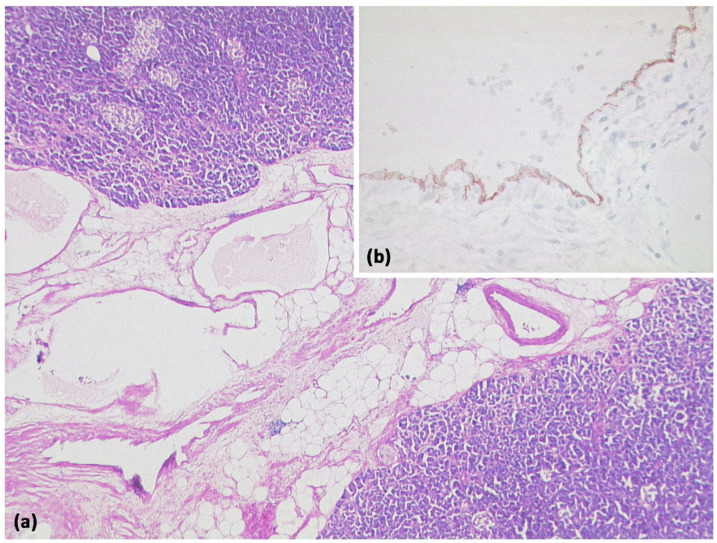
Lymphangioma ((**a**) HE, original magnification 25:1). Multicystic lesion in retroperitoneal adipose tissue and pancreas with cavities lined by a flat cell layer. The lining cell layer expresses the vascular marker CD31 ((**b**) original magnification 200:1).

Another rare subtype is cystic lymphangioma of the pancreas, which is considered a congenital malformation of the lymphatic system [50]. It results in the formation of multilocular cystic lesions, which are considered indolent and are usually discovered incidentally. A clustered occurrence in women has been described [50]. Some of these can reach enormous sizes of more than 10 cm and can also be palpable as a mass. On imaging, they are sharply delineated multilocular lesions, often with KM enhancement of the capsule or thin internal septa (Figure 19). Rarely, phlebolithic calcifications may be present. On MRI, cyst spaces appear hypointense on T1-weighted images and hyperintense on T2-weighted images [51]. Any fine-needle aspiration shows nonspecific features (Figure 20) [50].

**Figure 21:**
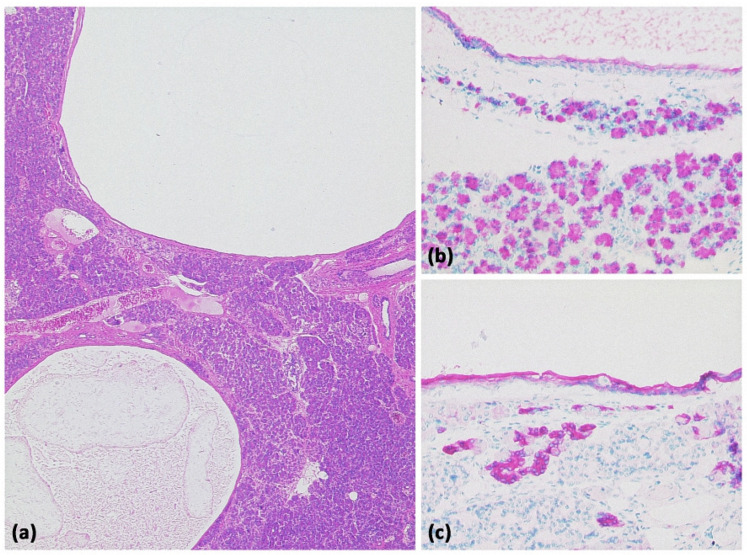
Acinar-cystic transformation of the pancreas with multiple intraparenchymal cysts ((**a**) HE, original magnification 25:1). Immunohistochemistry demonstrates partial acinar differentiation ((**b**) expression of trypsin, original magnification 100:1) and partial ductal differentiation ((**c**) expression of CK7, original magnification 100:1).

Acinar cell carcinomas of the pancreas are much less common than ductal adenocarcinomas or, for example, neuroendocrine neoplasms of the pancreas and account for only about 1% of all pancreatic neoplasms [52]. They are cell-rich neoplasms that, among other things, show acinar growth patterns and typically do not form desmoplastic stroma (Figure 21) [52]. Immunohistochemically, acinar differentiation can mostly be detected by demonstrating trypsin expression [52]. Mixed neuroendocrine differentiation may be present.

Endoscopic ultrasound (EUS) is recommended as an adjunct to other imaging modalities (grade 2C recommendation), although accurate subtype differentiation is not possible as with other modalities [7]. Specifically, EUS is recommended when malignancy-specific changes have been detected either clinically or radiologically. Data on the differentiation between benign and malignant neoplasms by ultrasound are inconsistent with considerable interobserver variability [7]. Further improvement can be expected from contrast-enhanced EUS (CE-EUS). Thus, the role of CE-EUS in the detection of mural nodules in cystic pancreatic lesions as well as areas suspicious for malignancy therein has already been investigated in several articles. In a recent meta-analysis, the value of CE-EUS in characterizing mural nodules in cystic pancreatic lesions was revealed [53]. Here, a special mode called CH-EUS (Contrast Harmonic-Enhanced Endoscopic Ultrasound) provides even higher diagnostic accuracy in the identification and characterization of mural nodules compared with conventional CE-EUS (pooled sensitivity 97.0% vs. 88.2% and specificity 90.4% vs. 79.1%, respectively).

Fine-needle aspiration complementary to imaging can be performed to differentiate mucinous from non-mucinous neoplasia if CT and MRI are discordant or unclear (grade 2C recommendation) [7]. In this regard, combined analysis of CEA and lipase levels from cyst fluid and cytology provide the highest accuracy for differentiating the underlying pathology (grade 2C, recommendation) [7]. However, an endoscopic ultrasound-guided biopsy of cystic lesions of the pancreas involves a non-negligible risk of complications. The technique should therefore be used selectively. For patient selection in order to optimize the benefit/risk ratio, a recent multicenter study is helpful [54]. Here, age, the number of biopsies, complete cyst aspiration, and diagnosis of IPMN were identified as independent predictors of adverse events at intervention. In the same work, a recursive partitioning analysis identified three classes of risk, with the highest risk in the case of IPMN and multiple biopsies [54].

## 5. Conclusions

A detailed and comprehensive radiological-morphological description of cystic lesions of the pancreas is essential for a correct image-morphological diagnosis and thus as a building block for the determination of the further procedure, in order to better assess the risk of malignant transformation or not to overlook already existing signs of malignancy. Knowledge of the most common histological entities and their clinical relevance is, therefore, of essential relevance in daily clinical practice. Equally important is the correct procedure, both in surgical therapy and regular follow-up. For this purpose, the European guidelines provide important guidance for a uniform and interdisciplinary coordinated approach.

## Figures and Tables

**Table 1:** Overview and classification of the most important pancreatic lesions [7,16,17,18,19].

Group	Tumors
Epithelial neoplastic	Mucinous • Intraductal papillary neoplasms • Mucinous neoplasms Non-mucinous • Serous cystadenoma • Serous cystadenocarcinoma • Cystic neuroendocrine tumors • Acinar cell carcinoma with cystic degeneration • Cystic ductal adenocarcinoma • Solid-pseudopapillary neoplasia • Cystic hamartoma • Cystic teratoma
Epithelial non-neoplastic	• Simple cyst • Congenital cyst • Lymphoepithelial cyst • Simple mucinous cyst • Retention cyst • Acinar-cystic transformation
Non-epithelial neoplastic	• Lymphangioma • Sarcoma
Non-epithelial non-neoplastic	• Pseudocyst • Parasitic cyst • Walled-of necrosis

## Data Availability

Not applicable.
